# Using patients’ own knowledge of early sensations and symptoms to develop an interactive, individualized e-questionnaire to facilitate early diagnosis of lung cancer

**DOI:** 10.1186/s12885-021-08265-x

**Published:** 2021-05-13

**Authors:** Adrian Levitsky, Britt-Marie Bernhardson, Ingela Henoch, Maria Olin, Karl Kölbeck, Nadja Rystedt, Carol Tishelman, Lars E. Eriksson

**Affiliations:** 1 Division of Innovative Care Research, Department of Learning, Informatics, Management and Ethics, Karolinska Institutet, SE-171 77 Solna, Sweden; 2 Cancer Proteomics Mass Spectrometry, Department of Oncology-Pathology, Karolinska Institutet, Science for Life Laboratory, SE-171 65 Solna, Sweden; 3 Sahlgrenska academy, University of Gothenburg, Institute of Health and Care Sciences, SE-405 30 Gothenburg, Sweden; 4 Lung Oncology Center, Theme Cancer, Karolinska University Hospital, SE-171 76 Solna, Sweden; 5 Center for Medical Technology and Radiation Physics, University Hospital of Umeå, SE-901 85 Umeå, Sweden; 6 Center for Health Economy, Informatics and Health System Research (CHIS), Stockholm Health Care Services (SLSO), Stockholm County Council, SE-113 65 Stockholm, Sweden; 7 School of Health Sciences, City, University of London, Northampton Square, EC1V 0HB, London, UK; 8 Medical Unit Infectious Diseases, Karolinska University Hospital, SE-141 86 Huddinge, Sweden

**Keywords:** Lung cancer, Respiratory diseases, Think-aloud interviews, Instrument development, Questionnaire design, E-questionnaire, Internet, Usability, Tablet computers, User-computer interface

## Abstract

**Background:**

One reason for the often late diagnosis of lung cancer (LC) may be that potentially-indicative sensations and symptoms are often diffuse, and may not be considered serious or urgent, making their interpretation complicated. However, with only a few exceptions, efforts to use people’s own in-depth knowledge about prodromal bodily experiences has been a missing link in efforts to facilitate early LC diagnosis. In this study, we describe and discuss facilitators and challenges in our process of developing and initial testing an interactive, self-completion e-questionnaire based on patient descriptions of experienced prodromal sensations and symptoms, to support early identification of lung cancer (LC).

**Methods:**

E-questionnaire items were derived from in-depth, detailed explorative interviews with individuals undergoing investigation for suspected LC. The descriptors of sensations/symptoms and the background items obtained were the basis for developing an interactive, individualized instrument, PEX-LC, which was refined for usability through think-aloud and other interviews with patients, members of the public, and clinical staff.

**Results:**

Major challenges in the process of developing PEX-LC related to collaboration among many actors, and design/user interface problems including technical issues. Most problems identified through the think-aloud interviews related to design/user interface problems and technical issues rather than content, for example we re-ordered questions to be in line with patients’ chronological, rather than retrospective, descriptions of their experiences. PEX-LC was developed into a final e-questionnaire on a touch-screen smart tablet with one background module covering sociodemographic characteristics, 10 interactive, individualized modules covering early sensations and symptoms, and a 12th assessing current symptoms.

**Conclusions:**

Close collaboration with patients throughout the process was intrinsic for developing PEX-LC. Similarly, we recognized the extent to which clinicians and technical experts were also important in this process. Similar endeavors should assure all necessary competence is included in the core research team, to facilitate timely progress. Our experiences developing PEX-LC combined with new empirical research suggest that this individualized, interactive e-questionnaire, developed through systematizing patients’ own formulations of their prodromal symptom experiences, is both feasible for use and has potential value in the intended group.

## Background

Lung cancer (LC) accounts for nearly 1/5th of the world’s estimated cancer-related deaths, as most individuals are diagnosed in advanced stages [1, 2]. One reason may be that sensations and symptoms potentially associated with LC are often diffuse [3–5] and expressed through a variety of words, thus complicating interpretation [4]. A wide range of diffusely formulated sensations and symptoms may delay care, as many might not be considered serious or urgent [3–5]. However, with only a few notable exceptions [5, 6], efforts to use people’s own in-depth knowledge about prodromal bodily experiences is a missing link in efforts to facilitate early LC diagnosis. One reason for this may be related to the difficulties inherent in differentiating among the complex clusters of often subtle sensations and symptoms which may indicate not only LC, but also a wide range of other conditions. In-depth descriptions of bodily experiences that might potentially be associated with LC are thus an important point of departure. Given the breadth of possibilities that each person’s symptomatic profile may provide, any initiative that seeks to systematize this wide variation would need a carefully-designed, user-friendly tool offering a wide range of expressive capacity. Thus, after obtaining qualitative data about early symptoms and sensations potentially associated with LC, the authors sought to develop a flexible, detailed but clinically-applicable instrument, capable of aiding in LC diagnostic investigation to fill this gap.

Pen and paper questionnaires were once praxis, however, e-questionnaires can in some cases be more feasible today [7–9]. Paper questionnaires, for example, require resources to transfer data to an electronic form for analysis. Both forms can require several rounds of testing and improvement, resulting in considerable time constraints before being ready to use [7–10]. Both demand a range of expertise for their design; they need to be user-friendly, contextually valid and relevant, and minimize participant burden as much as possible, while maintaining reliability in collecting required information [8]. However, while sharing some start-up challenges with paper questionnaires, e-questionnaires have other development costs, especially related to the IT competence needed to develop and maintain them and fix technical errors. In clinical practice, especially for those with an encumbering disease like LC, e-questionnaires that can be individualized through interactivity may be one way to minimize burden for patients [11].

We therefore designed an individualized, interactive e-questionnaire for in-depth investigation of early experiences of sensations and symptoms experienced by patients referred for suspected LC. The aim of this study is thus to describe and discuss facilitators and challenges we encountered in the process of developing and initial testing of this e-questionnaire, with content based on patients’ experiences, to facilitate future endeavors of this sort.

## Methods and results

This study was approved by the regional ethics board (EPN: #2008/51–31/3; #2011/438–32). The project derived from common interests shared with a UK research group [4], but was itself an independent multi-regional Swedish initiative led by nurse researchers. We worked with key actors, including representatives from the Swedish lung cancer patient association, clinical nursing and medical staff, and statisticians/epidemiologists.

**Fig. 1:**
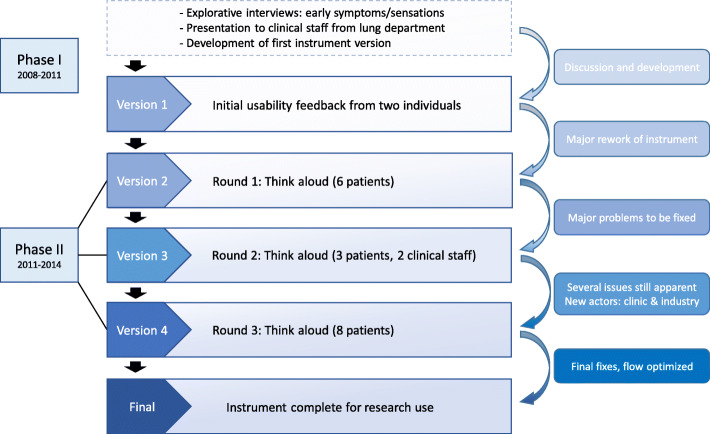
Developing the Patient EXperience of Bodily Changes for Lung Cancer (PEX-LC) e-questionnaire

The full process of developing the Patient EXperience of Bodily Changes for Lung Cancer (PEX-LC) e-questionnaire included three rounds of think-aloud interviews, illustrated in the flow chart, Fig. 1.

### Phase I

At the onset of this project (Fig. 1), early descriptors of sensations and symptoms were derived from explorative interviews carried out in several regional Swedish hospitals, with 60 patients undergoing evaluation for suspected LC (subsequent diagnosis: LC = 31; not LC = 29). Participants were asked about precursors of their present contact with the healthcare system and were explicitly asked about the knowledge they have about their own bodily experiences. We probed for in-depth descriptions of those early sensations and symptoms patients recalled. These open, volunteered descriptions were complemented with questions checking the presence and descriptions of other well-known LC symptoms. Background information (i.e. sociodemographic characteristics, smoking status, and comorbidities) was also elicited. The interviews were audio-taped and transcribed verbatim, and these data were analyzed inductively using NVivo software. The nearly 300 descriptors and background variables derived in this manner were the foundation for the development of the PEX-LC e-questionnaire.

To be able to individualize documentation of patients’ experiences of their own early sensations and symptoms, we concluded that an interactive e-questionnaire would need to be developed to allow each person to respond to items tailored to and appropriate for their specific symptomatic profile. The development of PEX-LC was made possible through collaboration with IT consultants.

Prior to development of the first version of PEX-LC, we used the wording from the interviewed individuals to create descriptors of early sensations and symptoms. We then initiated a process of meticulously merging descriptors to create questionnaire items related to different symptomatic areas. Challenges were encountered in the merging process, as different individuals used different words for similar phenomena (e.g. “in pain” vs. “hurting”); we therefore made efforts to differentiate between potentially distinct sensation qualities and words used as synonyms. Also, we were aware of the risk of removing a rare symptom which might be highly predictive.

At this time, our intentions for the e-questionnaire were to: 1) use patients’ wording for descriptors, considering potential regional/dialectical differences, 2) formulate as few relevant items as possible, tailored to the individual to reduce the burden of responding to the questionnaire, 3) begin by asking about the individual’s current state of health, followed by items about the initial experience of the particular problem, 4) complement these data with scale-based questions assessing current symptom intensity, and 5) summarize questionnaire responses upon completion for clinical use. The merged and reformulated descriptors and background variables were presented to lung medical and nursing clinicians to check face validity and get professional feedback.

### Version 1 of PEX-LC

The first PEX-LC prototype was developed with IT consultants according to the points outlined above and then tested in 2011 with two purposefully-chosen individuals known to the researchers, to obtain different types of user feedback on PEX-LC’s feasibility and usability. One individual was chosen from the general public, while the other was a patient activist expert on IT usability. Their responses confirmed our suspicion that Version 1 was an excessively long questionnaire that required a more feasible structure prior to use. As a result, the scale-based questions assessing symptom intensity were removed, as they were peripheral to our aim.

### Phase II

As seen in Fig. 1’s overview, during Phase II the e-questionnaire was tested and revised in three rounds of think-aloud interviews [12, 13] for versions 2–4 of PEX-LC, respectively. During this time contracted IT consultants assisted with the development of PEX-LC, including adapting layout and addressing technical issues through several versions. Statisticians worked with the consultants to ensure the accessibility of relevant information in quantifiable terms.

### Version 2 of PEX-LC

Based on the feedback described above, a second version of PEX-LC was developed and tested in a first round of think-aloud interviews with six patients with LC.

The think-aloud technique entails one-to-one cognitive interviewing during which participants are asked to verbalize their thoughts and feelings as they complete the questionnaire, to be able to gain insight into ideas, interpretations and cognitive processes at play as they arrive at a response. Think-aloud aims to ensure face validity of a tool, as it can identify the extent to which the instrument is understood as intended and help to resolve potential misunderstandings [13, 14]. It thus may maximize the performance of the questionnaire through a step-by-step quality check through the user’s verbalized cognitive processes, i.e. how the user comprehends, retrieves, judges, and responds to information [13]. Individuals were also asked to reflect during and after completion what they liked or disliked when using the e-questionnaire. Brief probing questions were used concurrently to better understand the basis for responses, while distracting as little as possible to minimize task flow disruption [12, 15].

A number of major problems related to content and technical aspects became clear through these think-aloud interviews. For example, those questions that were unclearly phrased became more apparent. We noted problems for patients in differentiating temporal aspects, e.g. reporting having a symptom at present versus when it first manifested. We rephrased these types of questions for the next version and emboldened the font to help distinguish present versus earlier symptoms.

We also noted a technical problem on the timeline used for patients to indicate when sensations and symptoms first began, which further compounded difficulties in reporting temporal aspects. Even other technical issues were found which led to a redesigning of PEX-LC, requiring further collaboration with IT consultants.

### Version 3 of PEX-LC

With the third version of PEX-LC, a new round of think-aloud interviews was carried out with three patients with LC and two clinical staff. Both a laptop and smart tablet version were tested in this round, leading to a decision to use the questionnaire in tablet form as it was both more user-friendly and easier to handle in the clinical setting, regardless of patients’ computer experience. However, many usability issues, including technical bugs, were still encountered. For example, respondents still had difficulties in completing the questionnaire due to technical layout problems. The temporal issue in Version 2 had not been resolved; despite our efforts, it was still difficult to distinguish current versus earlier symptoms. The new tablet layout also led to confusion between the answering field and the scrolling area.

As we resolved some issues, new problems became visible through the think-aloud interviews, i.e. lack of consistency in response alternatives and highlighting colors. We also noted the importance of appropriate layout for users to be able to understand when additional probing questions were linked to a specific descriptor. When we tested PEX-LC in a relevant clinical setting, we also encountered unexpected problems with hospital internet access, which led to a need to test different internet providers for the e-questionnaire, as hospital data security and ethical guidelines precluded our connection to the hospital network.

A number of important suggestions came from participants in the think-aloud interviews, e.g. adding a progress bar to PEX-LC to gauge remaining questions. An introductory title and brief summary were also suggested for the module overview page, for better orientation.

Overall, through this process, we were able to improve usability through a more streamlined questionnaire with better aesthetic appeal. However, several issues highlighted led to a need for major technical reworking and further instrument enhancement. There was a change of IT consultants during this period, which led to long delays and the need to reorient new individuals to our underlying goals and the work to date. One new IT consultant began to work in closer collaboration and more proximity with the research group. This helped resolve issues in a more timely fashion, as longer distances had created delays with continuity and distance could hinder understanding.

In summary, based on the think-aloud interviews and better contact with IT consultants, we undertook a major rework of PEX-LC on several levels. We simplified the layout through removal of redundant questions and updated the Background module to include potentially relevant comorbidities, as we recognized the difficulties for patients in differentiating their experiences by diagnosis; we therefore needed more clarity about their full situation. To further improve PEX-LC based on participants’ input, we discarded our original intention of having a retrospective approach, first asking about current state of health, followed by questions related to when problems began. Instead, a chronological approach was implemented as this seemed to fit better with participants’ ways of recalling; early sensations or symptoms were now addressed first, followed by questions about more recent changes in experienced phenomena.

We had also prepared the e-questionnaire to summarize patients’ response profiles which would be printed and made available for clinical staff, to facilitate communication about patients’ symptom experiences. However, in addition to technical issues, we now found that staff did not perceive the summary to be as relevant as initially suggested, as they had begun to use an internal symptom questionnaire. We instead determined in collaboration with clinicians to use an additional validated module focusing on current health. We tested several and together chose the European Organization for Research and Treatment of Cancer Quality of Life Core Questionnaire (EORTC-QLQ-C30) [16] as a 12th module in PEX-LC.

At this point, we began to hold more frequent joint meetings of the research group and clinicians at the lung medicine department, to begin further planning and protocol development for pre-diagnostic recruitment for a later clinical study with PEX-LC. A nurse researcher working at the lung medicine department was hired, to facilitate closer collaboration with the clinical setting.

### Version 4 of PEX-LC

The last round of think-aloud interviews was carried out with eight patients with LC, testing the fourth version of PEX-LC. The interactivity of the e-questionnaire was encouraging and no further major reworking was deemed necessary, as most issues appeared to have been successfully resolved. We also noted that the patients seemed to have no trouble in selecting descriptors that suited their individual experiences, which suggested that PEX-LC’s content adequately reflected the target group’s sensations and symptoms.

Some minor issues, however, were still apparent. Problems with internet and mobile signals at the hospital remained, and it was still necessary to use a mobile internet connection due network issues beyond our control. Finally, problems with the amount of time required to complete the questionnaire in a busy hospital setting were noted as we began to develop the protocol for a larger clinical study. Thus, in addition to fixing minor bugs, instructions were adapted to optimize use of patients’ limited time.

### Final version of PEX-LC

PEX-LC was developed into a final e-questionnaire with 12 interactive modules on a touch-screen smart tablet, allowing assessment of a symptom from its’ first manifestation until present time.

**Fig. 2:**
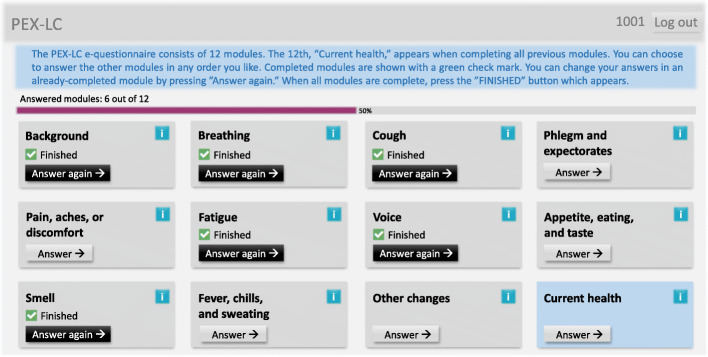
Finalized interactive modular overview of the Patient EXperience of Bodily Changes for Lung Cancer (PEX-LC) e-questionnaire

Figure 2 illustrates the finalized interactive modular overview of PEX-LC. A progress bar indicates number and percentage of overall module completion, and completed modules are indicated with green check marks. Information boxes for each module, when tapped, provide brief background information about the questions in that module. Modules do not have to be completed sequentially but can be responded to in the order the participant chooses.

The final e-questionnaire, as shown in Fig. 2, includes 11 base modules: Background; Breathing Difficulties; Cough; Phlegm/Expectorates; Pain/Aches/Discomfort; Fatigue; Voice Changes; Appetite/Eating/Taste Changes; Olfactory Changes; Fever/Chills/Sweating; and Other Changes (e.g. general physical health, malaise) – with the additional 12th module focusing on health status during the past week. The 11 base modules contain a total of 484 possible items, consisting of background questions and in-depth descriptors of early sensations or symptoms, including follow-up items. However, the e-questionnaire is individualized in response to the participant’s particular state of health. Therefore, not all follow-up questions are seen by each participant, as only those questions which are relevant in relation to each individual’s previous response profile are shown.

**Fig. 3:**
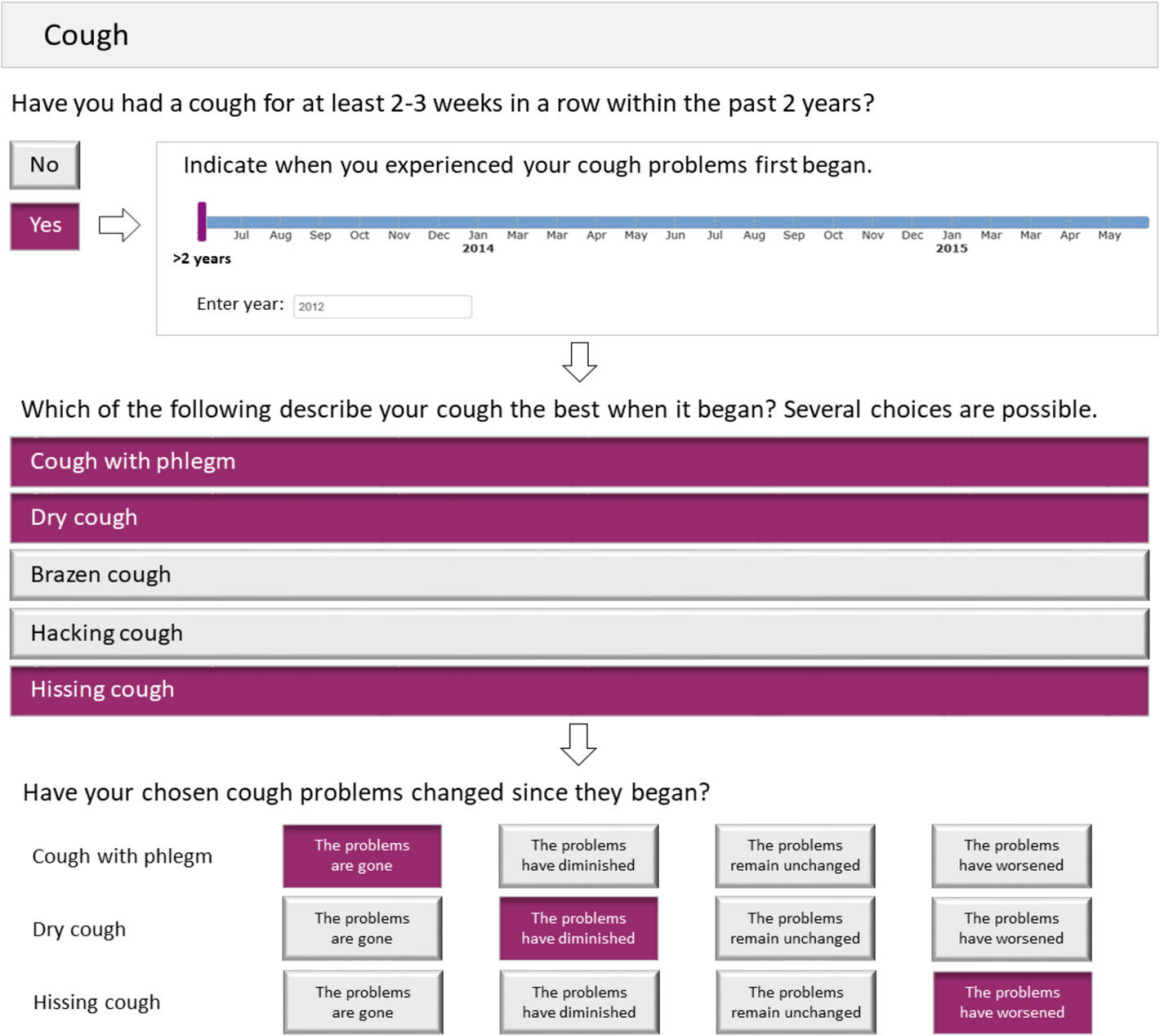
Cough module flow example from the Patient EXperience of Bodily Changes for Lung Cancer (PEX-LC) e-questionnaire

**Fig. 4:**
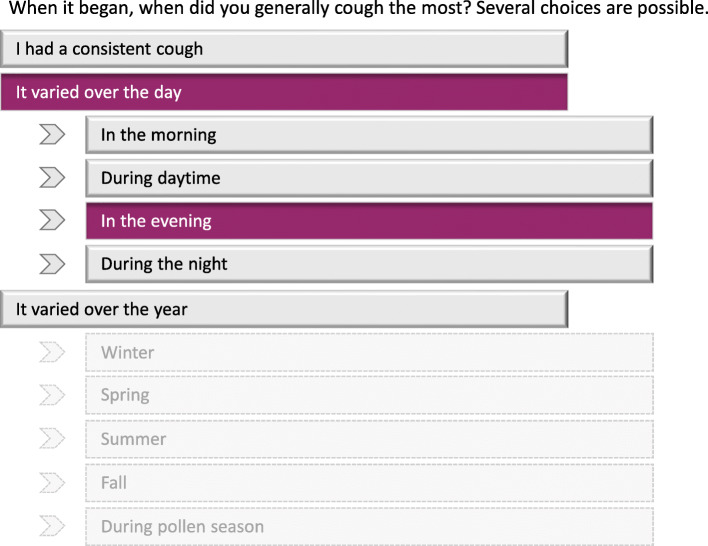
Flow example of follow-up descriptors in the Patient EXperience of Bodily Changes for Lung Cancer (PEX-LC) e-questionnaire

The interactivity of the e-questionnaire is illustrated in Figs. 3 and 4 using an example from the Cough module. The first question in Fig. 3 appears on the screen at the start of the module. Should the user answer “Yes,” s/he then progresses to the timeline bar, where the month and year the sensations or symptoms first began can be indicated. If the sensation first appeared over 2 years ago, the user would type in the year it was first noticed. The last image in Fig. 3 illustrates follow-up questions, with several choices of descriptors possible. Figure 4 shows an example of the type of follow-up descriptors which appear after some responses. Here, “It varied over the day” was first selected, with the follow-up response, “In the evening” chosen among several possible follow-up descriptors. Note that follow-up descriptors are indented for clarity on the e-questionnaire; the follow-up descriptors (shown in gray) would not be seen unless the original corresponding descriptor was chosen.

## Discussion

In this article, we describe the development of an interactive e-questionnaire, PEX-LC, designed to aid in diagnostic investigation of early sensations and symptoms of possible LC. Initial interviews with 60 patients under investigation for suspected LC gave rise to all descriptors and most background variables in PEX-LC. Initially, together with the research team, IT consultants focused on developing the technical aspects of the instrument in relative isolation; however, during the development process we recognized the importance of better integrating the IT consultants into the research team. A series of think-aloud interviews led to the refinement of the e-questionnaire through several versions and pointed to the need for better integration of content and technical aspects.

In accordance with our original intentions in developing PEX-LC, we maintained colloquial wording of descriptors, and diminished participant burden by individualizing interactivity, using as few relevant questions as possible for each specific respondent. We, however, adapted our original retrospective approach for several reasons, most importantly that the think-aloud interviews made clear that people tend to think chronologically, rather than retrospectively. Thus, PEX-LC was re-designed so that the earliest problems that surfaced were first described, with further probing about later relevant changes. Additionally, scale-based questions for symptom intensity were removed after initial testing to avoid overburdening users, and became superfluous as current state of health was covered by PEX-LC’s follow-up questions and the EORTC-QLQ-C30, which also assesses symptom intensity. Finally, the planned summary of the questionnaire was omitted due to perceived lack of clinical relevance.

Previous studies have identified several early LC-associated signs and symptoms [5, 17–20], including hemoptysis, dyspnea, cough, and/or weight loss, and in some instances, changes in appetite [5, 17, 18] and fatigue [5, 17], up to 2 years before diagnosis. While PEX-LC also includes these symptoms, we are able to assess them in depth with more detail, and they have been complemented with less common symptoms. While all data about patient experiences in these extant studies are retrospective, most of them derive from general medical records and/or were obtained post-diagnosis. However, data from medical records means that patient experiences are filtered by professionals, and knowledge of diagnosis and treatment side-effects may create biases in patient recall. To our knowledge, only one other study surveyed individuals referred for LC investigation prior to diagnosis as is intended with PEX-LC, in that case using a brief questionnaire with 20 symptoms [6]. To summarize, two unique features of PEX-LC are that all descriptors are derived from open, detailed interviews with patients about their experiences, and that this data was obtained prior to rather than post-diagnosis.

Several key issues needed to be addressed before the PEX-LC e-questionnaire could be used in clinical research settings. The most substantial challenges encountered in developing PEX-LC are discussed below, and were related to collaboration among actors in the development process; design/user interface problems and technical issues; and clinical usability.

### Collaboration among actors in the development process

We collaborated with three main groups of actors in developing PEX-LC: patients, clinical staff, and IT consultants. A major strength of the development process and e-questionnaire itself is that patients and their knowledge about their own bodies and situation were crucial throughout. This was facilitated through our long-term contact with the Swedish LC patient association and with different lung medicine hospital departments, and by using think-aloud interviews. Patients willingly shared their substantial knowledge about their experiences with us, thus making PEX-LC more comprehensive and relevant than instruments based only on professional perspectives. At no point in PEX-LC’s development did any participant mention experiencing a sensation or symptom not already included. We also received feedback that each patient’s unique symptom profile could be expressed through the available response options. The comments and issues raised by patients were instead most often related to the technical interface.

Through our collaboration with IT consultants, usability and technical issues were eventually resolved, although the e-questionnaire development took a considerable length of time. The time taken to resolve technical issues was in part due to changes in IT consultants, communication problems with consultants who did not work closely with the research group, and to some degree, a lack of relevant IT expertise among the core research team, leading to our inability to adequately anticipate and sometimes communicate our IT needs. A major facilitator in finalizing PEX-LC was a closer—both geographically and in terms of teamwork—collaboration with the last IT consultant.

Collaboration with clinical staff was periodic throughout the study. There was a need to maintain enthusiasm in all stages of the development process, with continued reaffirmation and contact. Occasional passive resistance from staff and middle-managers, in different stages of PEX-LC’s development, caused delays in implementing decisions made in collaboration between senior clinicians/managers and researchers. To ensure the facilitation of clinical collaboration on all levels, we began to arrange regular meetings with clinical nursing staff, and employed a clinical nurse in the team to facilitate contact with the clinical staff and help carry out the study.

In retrospect, drawing from knowledge gained through PEX-LC development, we suggest that similar processes might be optimized if all types of stakeholders are active in the research team from study conception.

### Design/user interface problems and technical issues

Interface challenges were encountered throughout PEX-LC’s development. In designing the earliest versions, we did not recognize that ordering questions chronologically instead of retrospectively would be more in line with natural thought processes. This points to the importance of continual input from potential respondents in optimizing usability. This input was also important in understanding and responding to a wide variety of technical issues. Had we discussed the issues earlier, clinicians might have made us cognizant of the ethical and legislative boundaries we later encountered, which limited internet access in the intended setting for clinical research. Better IT and design competence in the research team might have helped heighten usability and resolve technical issues and bugs in a timelier fashion. Again, this motivates having a broader range of competencies working together in a core team.

### Clinical usability and validity

Lessons learned from the literature, including a prior development study, demonstrated that a smart tablet e-questionnaire was feasible for use in a palliative clinical setting [21], which is a group with a profile relatively similar to those individuals with potential LC who are targeted by PEX-LC. Additional smart tablet questionnaire developments in health-burdened individuals include Smaradottir et al.’s [22] telemedicine application to assess user-reported symptoms for individuals with chronic obstructive pulmonary disease. As in our study, they used think-aloud techniques. The design strengths noted in both their instrument and PEX-LC were similar, e.g. appropriate color contrast and main screen interactivity, as were challenges, e.g. the need to include a progress bar and to resize touch buttons [22]. However, Smaradottir et al.’s [22] study used an instrument with an interface that demanded more IT familiarity from users. Nonetheless, the respondents who tested that smart tablet questionnaire found it usable.

Despite the many issues made apparent through repeated rounds of think-aloud interviews regarding design and interface of our smart tablet e-questionnaire, in its’ final, individualized form with a wide array of possible descriptors, PEX-LC was able to be successfully implemented in a large-scale clinical study with data collected from 670 patients undergoing diagnostic work-up for potential LC [23]. Despite the heavily health-burdened users in this group, who are often considered difficult to recruit, there was a high level of participation, suggesting perceived relevance for patients.

The inductive approach that gave rise to a breadth of individualized descriptors of PEX-LC managed to reflect the clinical complexities often leading to late diagnosis. Inclusion of the wide variety of early, pre-diagnostic patient symptom experiences allowed us to filter out and, using machine learning, determine predictors of primary LC based on data from a large subset of our cohort (506 individuals) [23]. We were able to identify 63 early symptoms and sensations and seven background variables in PEX-LC that were key to predicting primary LC. It is, however, important to continue to test the instrument in a larger general population to determine if the same variables are identified. It is promising that recently-published follow-up results from the nationwide population-based Danish Symptom Cohort [24], presenting a short set of nine investigated potential lung cancer alarm symptoms among 37,455 randomly-selected eligible individuals aged ≥40 [25] confirmed and cited some of our key identified LC risk symptoms and sensations, most notably appetite loss. These confirmatory findings are thus one step forward in confirming the general validity of PEX-LC. Better determination of PEX-LC’s clinical validity is however, also needed if we are to flag patients in time for early diagnostic workup.

Another issue for continued research is testing against other questionnaires to examine if PEX-LC provides data beyond that of other self-report instruments. Our intention was that PEX-LC would add granularity and nuance to self-reported data about symptom experiences beyond that generally assessed by less comprehensive questionnaires. We have plans to test PEX-LC against other existing instruments, however, some challenges in such comparison should be recognized. These include different timeframes, formulation of questions and symptoms assessed. However, a major challenge for comparison is that the interactive nature of PEX-LC means that the battery of questions answered differs by participant whereas it is constant in other instruments.

## Conclusions

In summary, close collaboration with patients throughout the process was intrinsic for developing PEX-LC. Similarly, we recognized the extent to which clinicians and technical experts were also important in this process. Similar endeavors should assure all necessary competence is included in the core research team, to facilitate timely progress. Both the experiences detailed in existing literature and our own experiences in developing and later successfully testing PEX-LC in our large clinical cohort suggest that this individualized and interactive e-questionnaire, developed through systematizing patients’ own formulations of their prodromal symptom experiences, is feasible for use in the intended group.

PEX-LC has potential to provide an important complement to other LC questionnaires, which are generally intended to assess current status, typically after diagnosis, by assessing early sensations and symptoms prior to diagnosis, while not affected by treatment. If its’ potential is realized after further testing for generalizability, it might provide new data to contribute to shortened time spans to LC diagnosis.

## Data Availability

The datasets used and/or analysed during the current study are available from the corresponding author on reasonable request.

## Abbreviations

LC: Lung cancer
PEX-LC: The patient experience of bodily changes for lung cancer
EORTC-QLC-C30: The European Organization for Research and Treatment of Cancer - Quality of Life Questionnaire - Core questionnaire
